# Synergistic Effect of Elastic Stockings to Maintain Volume Losses after Mechanical Lymphatic Therapy

**DOI:** 10.1155/2014/430636

**Published:** 2014-06-19

**Authors:** José Maria Pereira de Godoy, Renata Lopes Pinto, Ana Carolina Pereira de Godoy, Maria de Fátima Guerreiro Godoy

**Affiliations:** ^1^Cardiology and Cardiovascular Surgery Department, Medicine School in São José do Rio Preto (FAMERP), Avenida Constituição 1306, 15025-120 São José do Rio Preto, SP, Brazil; ^2^Research Group Godoy Clinic, São José do Rio Preto, Brazil; ^3^Medicine School of ABC, São Paulo, Brazil; ^4^Medicine School in São José do Rio Preto (FAMERP), São José do Rio Preto, Brazil; ^5^Post-Graduate Specialization Course on Lymphovenous Rehabilitation (FAMERP), São José do Rio Preto, Brazil

## Abstract

The objective of the current study was to assess whether Venosan elastic stockings have a synergistic effect on the maintenance of results after Mechanical Lymphatic Therapy. Eleven patients with grade II lymphedema of the legs, regardless of cause, were evaluated in the Clinica Godoy between September and November 2012. The participants' ages ranged from 53 to 83 years old with a mean of 65.1 years. Two groups were formed with Group I using Venosan elastic stockings and Group II not using any type of compression therapy. Evaluations of the lymphedematous legs were performed before and after each drainage session using bioimpedance. Patients who wore elastic stockings had a greater volume reduction than those who did not wear stockings (unpaired *t*-test: *P* value < 0.001).

## 1. Introduction

Lymphedema is an accumulation of water, salts, electrolytes, high molecular weight proteins, and other elements in the interstitial space resulting from dynamic or mechanical changes of the lymphatic system which lead to a progressive increase in size of an extremity or body region with decreased functional and immune capacity and morphological changes [1]. Clinical staging takes into account the manifestation of the edema and the deformities observed. In grade I lymphedema, the swelling appears during the day and in grade II, the patient awakens with edema in the morning which normally worsens during the day. Grade III lymphedema is similar to grade II but more advanced and with worse deformities [2]. Severity may be mild with a volume increase of up to 20% (compared to the normal contralateral leg), medium with increases of between 20% and 40%, or severe with increases of more than 40% [1, 2].

An association of therapies is recommended to treat lymphedema with lymph drainage, compression mechanisms, and exercising constituting the cornerstone of treatment [3–6]. Management of lymphedema using drugs is also possible. The RAGodoy device, a Mechanical Lymphatic Therapy option that uses plantar flexion and extension movements, is a new addition to the armory of lymphedema treatment [7, 8]. Several studies have shown its effectiveness in reducing the volume of legs [7]. The association of therapies is a clinical option, but it is essential to assess whether combinations provide a synergistic effect.

Compression is a physical force that, when applied to the skin using elastic or nonelastic materials, exerts a pressure on the internal tissues of the body, including the microcirculation and macrocirculation structures, resulting in decreases in edema and improvements in the functioning of limbs [9, 10]. The objective of the current study was to assess whether Venosan elastic stockings have a synergistic effect on the maintenance of the results achieved by Mechanical Lymphatic Therapy.

## 2. Method

### 2.1. Design

Eleven female patients were evaluated in a randomized prospective clinical study that assessed the use of elastic stockings to maintain the results achieved by Mechanical Lymphatic Therapy performed for two hours daily to reduce the swelling of lymphedematous legs. The volume of the lymphedematous limbs was assessed using bioelectrical impedance before and after Mechanical Lymphatic Therapy and after the use of elastic stockings for maintenance between therapeutic sessions.

### 2.2. Patients and Location

Eleven female patients with grade II bilateral lymphedema of the legs from the Clinica Godoy were enrolled in this study between September and October 2012. Their ages ranged between 53 and 83 years old (mean: 65.1 years).

The inclusion criteria were grade II lymphedema, regardless of cause, as evidenced by the formation of pitting (Godet sign) but without evidence of advanced fibrosis. Patients with limited joint mobility, allergy or intolerance to elastic stockings, and infection and those without an evident Godet sign were excluded.

### 2.3. Development

All patients were submitted to Mechanical Lymphatic Therapy using the RAGodoy device for two hours per day over two weeks. Consequently they were randomly allocated to two groups by lottery: Group I (*n* = ten legs) used 20/30 Venosan elastic compression knee-length stockings between mechanical lymph drainage sessions and Group II (*n* = twelve legs) did not use any type of compression mechanism. Mechanical Lymphatic Therapy uses an electromechanical device to perform passive movements with flexion and extension of the foot [7]. The treatment sessions were daily for two weeks and differences in leg volumes were measured at the start and end of each Mechanical Lymphatic Therapy for both groups of patients.

Bioimpedance was used to calculate the volume of the limbs before and after each drainage session using the S10 InBody Body Composition Analyzer (BioSpace, Seoul, Korea).

The unpaired *t*-test was used for statistical analysis with an alpha error of 5% (*P* value < 0.05) being considered acceptable. The study was approved by the Research Ethics Committee of the Medicine School in São José do Rio Preto (FAMERP) number 58460-107-2012.

## 3. Results

**Figure 1:**
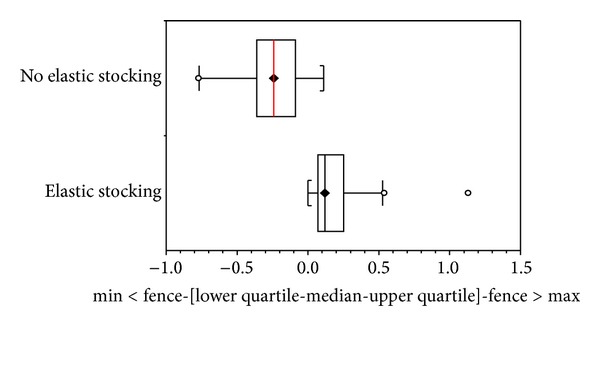
Box whisker plot showing leg volume changes between Mechanical Lymphatic Therapy sessions comparing patients who used elastic stockings with those without any type of compression.

Volume reductions were observed in all drainage sessions, but small gains occurred in all patients between sessions. However, a better maintenance of the volume reduction was observed in patients who wore elastic stockings than those who did not use any type of compression mechanism (unpaired *t*-test: *P* value < 0.001). The leg volume changes between Mechanical Lymphatic Therapy sessions comparing patients who used elastic stockings with those without any type of compression can be seen in Figure 1.

## 4. Discussion

The present study demonstrates the synergetic effect of the use of elastic stockings between Mechanical Lymphatic Therapy sessions to maintain volume reductions of lymphedematous legs. An association of therapies is recommended for the treatment of lymphedema [1]; however, an evaluation of the synergistic effect of each combination is not always emphasized and there are very few publications reporting specific evaluations.

It has been observed that patients with venous insufficiency who wear elastic stockings maintain, to a great extent, the volume losses that occur at night during all the day [10]. It is common during the course of the day for an increase in volume to come about; this is normal due to the effects of gravitational pressure [10, 11]. With the use of elastic stockings, the increase in volume is within the variations of the physiological reserve.

In grade II lymphedema, the swelling persists even with rest and so the aim of treatment is to reduce the edema. An association of therapies is recommended to treat lymphedema with lymph drainage, compression mechanisms, and exercises constituting the mainstays of treatment. Nonelastic mechanisms (bandages) are recommended but require specialized professionals to apply them. Thus, elastic stockings are an option that helps to maintain the results of lymph drainage or exercises.

The limitation of elastic stockings is the fact that their effectiveness requires constant evaluation; as the volume of the limbs reduces, the elastic stockings will cease to perform their function. Hence, they must be reassessed and when necessary changed or the overlapping of stockings should be used.

Recent studies show that it is possible to reduce most lymphedema to near normality [12]. The normalization of lymphedematous legs is important as it allows patients to buy stockings that exist in the market. Even so, it is important to remember that lymphedema has no cure and an association of lymph drainage is important in the lives of these patients.

Higher compression is recommended when choosing stockings and it is necessary to carefully follow up patients to assess volume losses or gains. In the daily practice it is common to see that when stockings lose their elasticity, the leg starts to swell again.

## 5. Conclusion

Elastic stockings have a synergistic effect to maintain volume reductions achieved with lymph drainage.

## References

[1] de Godoy JMP, Andrade M, Azevedo WF (2011). IV Latin American consensus on the treatment of lymphedema. *Journal Phlebology and Lymphology*.

[2] Lee B, Andrade M, Bergan J (2010). Diagnosis and treatment of primary lymphedema. Consensus document of the International Union of Phlebology (IUP)-2009. *International Angiology*.

[3] Partsch H, Damstra RJ, Mosti G (2011). Dose finding for an optimal compression pressure to reduce chronic edema of the extremities. *International Angiology*.

[4] Stout N, Partsch H, Szolnoky G (2012). Chronic edema of the lower extremities: international consensus recommendations for compression therapy clinical research trials. *International Angiology*.

[5] Kerchner K, Fleischer A, Yosipovitch G (2008). Lower extremity lymphedema. Update: pathophysiology, diagnosis, and treatment guidelines. *Journal of the American Academy of Dermatology*.

[6] Mosti G, Picerni P, Partsch H (2012). Compression stockings with moderate pressure are able to reduce chronic leg oedema. *Phlebology*.

[7] de Godoy JMP, de Fátima Guerreiro Godoy M (2004). Development and evaluation of a new apparatus for lymph drainage: preliminary results. *Lymphology*.

[8] Symvoulakis EK, Anyfantakis DI, Lionis C (2010). Primary lower limb lymphedema: a focus on its functional, social and emotional impact. *International Journal of Medical Sciences*.

[9] Cataldo JL, de Godoy P, de Barros J (2012). The use of compression stockings for venous disorders in Brazil. *Phlebology*.

[10] de Godoy JMP, Braile DM, Perez FB, de Fátima Guerreiro Godoy M (2010). Effect of walking on pressure variations that occur at the interface between elastic stockings and the skin. *International Wound Journal*.

[11] Belczak CEQ, de Godoy JMP, Ramos RN, de Oliveira MA, Belczak SQ, Caffaro RA (2010). Is the wearing of elastic stockings for half a day as effective as wearing them for the entire day?. *British Journal of Dermatology*.

[12] de Godoy JMP, Brigidio PAF, Buzato E, de Godoy MFG (2012). Intensive outpatient treatment of elephantiasis. *International Angiology*.

